# The Evolution of Polystyrene as a Cell Culture Material

**DOI:** 10.1089/ten.teb.2018.0056

**Published:** 2018-10-06

**Authors:** Max J. Lerman, Josephine Lembong, Shin Muramoto, Greg Gillen, John P. Fisher

**Affiliations:** ^1^ Department of Materials Science and Engineering, University of Maryland, College Park, Maryland.; ^2^ Surface and Trace Chemical Analysis Group, Materials Measurement Lab, National Institute of Standards and Technology, Gaithersburg, Maryland.; ^3^ NIH/NIBIB Center for Engineering Complex Tissues, University of Maryland, College Park, Maryland.; ^4^ Fischell Department of Bioengineering, University of Maryland, College Park, Maryland.

**Keywords:** polystyrene, surface chemical modification, plasma treatment, custom fabrication, electrospinning, 3D printing

## Abstract

Polystyrene (PS) has brought *in vitro* cell culture from its humble beginnings to the modern era, propelling dozens of research fields along the way. This review discusses the development of the material, fabrication, and treatment approaches to create the culture material. However, native PS surfaces poorly facilitate cell adhesion and growth *in vitro*. To overcome this, liquid surface deposition, energetic plasma activation, and emerging functionalization methods transform the surface chemistry. This review seeks to highlight the many potential applications of the first widely accepted polymer growth surface. Although the majority of *in vitro* research occurs on two-dimensional surfaces, the importance of three-dimensional (3D) culture models cannot be overlooked. The methods to transition PS to specialized 3D culture surfaces are also reviewed. Specifically, casting, electrospinning, 3D printing, and microcarrier approaches to shift PS to a 3D culture surface are highlighted. The breadth of applications of the material makes it impossible to highlight every use, but the aim remains to demonstrate the versatility and potential as both a general and custom cell culture surface. The review concludes with emerging scaffolding approaches and, based on the findings, presents our insights on the future steps for PS as a tissue culture platform.

## Introduction: How Polystyrene Became the Basis of *In Vitro* Cell Culture

Polystyrene (PS) has served as the fundamental substrate for adherent animal and human cell culture for >50 years.^1^ Due to its optical clarity, relative ease of manufacture, and low production cost, PS has largely replaced glass for cell-based work,^2,3^ whereas glass remains the choice for imaging due to its lower refractive index.^4^ On the other hand, mass production of PS through injection molding has produced a low-cost, high-culture volume, alternative to glass, which is compatible with many cell strains and contrast agents. All these reasons have driven two-dimensional (2D) tissue culture polystyrene (TCPS) to become the basic platform for adherent cell culture.

**FIG. 1.**
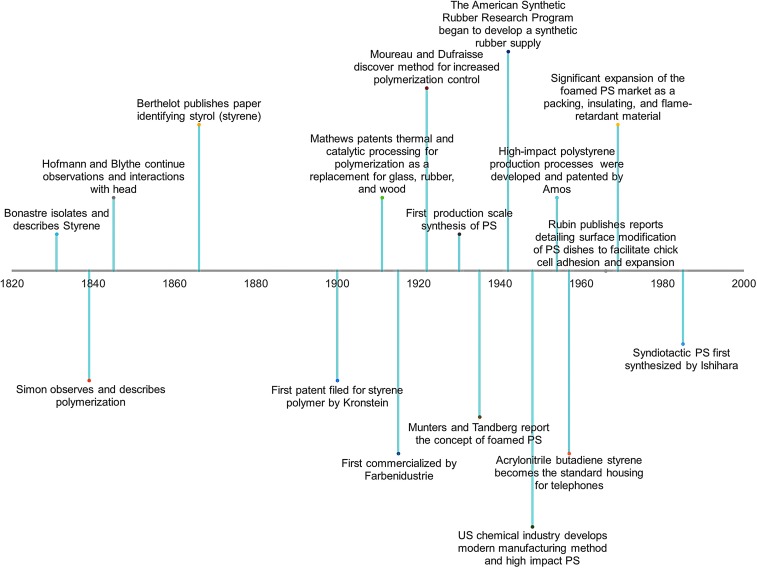
Timeline summarizing major developments of PS, from the initial discovery to custom-compounded polymers. Major development of the plastic occurred during and after World War II with the need for a consistent synthetic rubber supply. PS has played a pivotal role in many industries, with nearly 200 years of research attributed to this single material. PS, polystyrene.

**FIG. 2.**
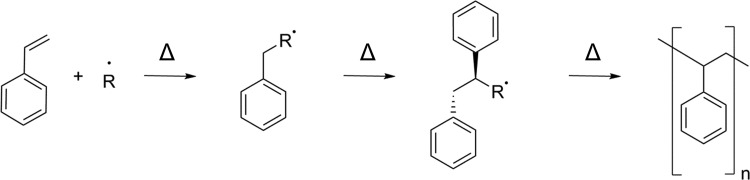
The free-radical polymerization process for PS synthesis. The free-radical incorporates into the styrene monomer and continues to increase the polymer length by breaking the *pi–pi* on the vinyl group, forming a new *sigma* bond.

PS development began in the 1830s with the discovery of styrene and the first documented observations of polymerization (Fig. 1).^5,6^ Development of styrene-containing polymers continued, with major advances occurring along with the advent of large-scale plastic processing, spurred by World War II.^6,7^ Modern applications bridge multiple industrial areas from cell culture to synthetic rubber, with material development constantly ongoing. Modern applications for PS harness the inherent material properties, largely as highly recyclable injection molded or thermoformed plastic, to achieve consumer and research goals.^5,8^ With a second-order glass transition temperature near the boiling point of water (95–105°C, with some molecular weight dependence),^9–12^ the formability of the material eases manufacturing constraints, both as a compounded and pure material. The use of PS in biomanufacturing and cell-based research activities as an injection-molded, embossed, cast, electrospun and, more recently, three-dimensional (3D) printed polymer can all be attributed to the business drive to mass-produce culture plastics and move away from glass. However, the simple homopolymer lacks appropriate surface chemistry for cellular recognition: phenyl groups do not readily provide anchoring points for cells as they are not normally expressed in the human body (Fig. 2).^1^ This has dictated the need to modify and develop PS-based surfaces to facilitate cell anchorage *in vitro* by incorporating surface functionality, which cells will bind to and grow on, a major theme of this review.

Five decades of 2D PS spearheading *in vitro* cell culture approaches has built a foundation of knowledge, understanding mechanisms to expand cells generally. This review focuses on the basics of 2D culture platforms and emerging 3D PS approaches researchers are embracing for cell culture, summarizing the mechanisms to transform PS surfaces to facilitate cell adhesion, growth, and *in vitro* expansion. We discuss the liquid, plasma, and next-generation treatment methods used to alter PS surfaces to improve cell growth and how these methods allow for the incorporation of moieties containing oxygen and nitrogen, thereby presenting surface chemistry for cells to anchor and grow. As the importance of 3D surfaces is becoming widely accepted, we dive into the role 3D PS fabricated through casting, electrospinning, and 3D printing, seeking to increase our understanding of cell growth *in vitro* and how the complex growth platforms can better replicate *in vivo* environments. As well, we present a focus on PS microbeads, and the void they fill as a suspended–adherent growth substrate. Overall, this review details the approaches garnered for PS as both a basic and targeted growth substrate.

## 2D PS: The Basis of *In Vitro* Cell Culture

Fabricating mass-produced flat and finely topographically detailed 2D tissue culture surfaces both employ the same basic technologies: casting, embossing, or molding. With the fewest technical and equipment challenges, industrial scale injection molding has served to fabricate parts with features traditionally at the hundreds of micron scale and above, where improvements continue to push these boundaries.^13,14^ Since the 1970s, injection-molded PS, followed by an oxygen plasma treatment, has remained the most prolific method for manufacturing TCPS culture surfaces.^15^ However, custom in-house injection molding equipment is generally unavailable to research groups and mass-produced TCPS may not possess surface characteristics of interest. This is not to say laboratory bench embossing is out of reach. Imprinting fine channels, through holes, and other microfluidic features have been demonstrated for cell-based assays, although it may require a 15-ton hydraulic press with heating plates capable of reaching 125°C.^16^ Such equipment may aide in embossing PS surfaces, but is not necessary to achieve anisotropic surface behavior. Using a silicon template, nanoimprinting has been used to align osteoblasts, finding that deeper grooves improved cell alignment along the channels, and increased migration rate along 150 nm grooves by a factor of 1.46.^17–20^ Hot embossing has been used to create many fine features, including pillars, grooves, and microwells, which successfully spatially segregated cells by patterning the surface chemistry and topography.^21^ As well, micropattern width has been seen to influence differentiation of human mesenchymal stem cells to vascular smooth muscle cells, where finer widths aid in cell alignment.^22^ As the cells adhere to the micropattern, internal mechanical stresses likely act to differentiate the cell, working to match features to function.^23,24^ However, while sequestering the cells with microchannels may be beneficial to guide cells, the reduction in cell spreading and change in morphology may also generate genetic abnormalities (such as forming micronuclei),^25,26^ making cell culture platforms with relevant length scales larger than the cells still more desirable. With this quality in mind, casting and embossing remain two of the more reproducible means to produce PS parts, especially on the industrial scale with injection molding equipment, and remain the method of choice for consumer and industrial parts. The major drawback to casting is the prohibitive cost of the tooling and equipment, limiting the most practical uses to large volume part production. On the laboratory bench, the lack of specialized tooling or standardized technique can result in poor part resolution and high cycle times. Other fabrication methods, such as electrospinning and 3D printing, provide alternate ways to achieve resolution that cannot be accomplished by casting and embossing with the advantage of developing complex 3D structures. Following fabrication, PS must be surface treated to facilitate cell adhesion. Over time, these approaches have evolved and are discussed next.

## The Transformation of PS to TCPS

With respect to *in vitro* cell growth, biocompatible surfaces need to incite cell adhesion, spreading, and potentially induce differential cell function, based on the application. To facilitate cell adhesion, the PS surfaces are functionalized to introduce biologically relevant chemistry (e.g., carbonyl and amine groups). Transforming native PS surfaces to include chemistry other than phenyl groups can increase the hydrophilicity and surface charge, modulating the deposition of extracellular matrix, cells, and proteins.^27–29^ The complex mechanisms for cell deposition in *in vitro* models warrants further investigation, potentially developing a means to develop custom growth surfaces, therefore promoting large expansion of cells. As researchers seek to develop active linking mechanisms, growth surface functionality continues to evolve, sequestering specific cell types and using material properties to modulate adhesion.

Functionalization methods can be broadly divided into two groups: liquid phase and plasma-based treatments. Liquid treatments provide an easy avenue to treat large surfaces quickly, but the functionality gained is often limited to surface oxidation and often requires highly corrosive substances. Plasma functionalization, most commonly used to manufacture TCPS in bulk, broadens the surface chemistries achievable, but requires ionizing energy to modify the surface (which can pose some safety concerns as well). Emerging surface modification techniques provide targeted cell interaction mechanisms, by grafting specific binding regimes, such as DNA and proteins, but may have long-term stability issues and higher costs limiting their broad appeal. Discussion of each of these techniques, along with treatment fluid choices will address mechanisms for depositing specific chemistry, modulating contact angle, and roughening surfaces to facilitate cell adhesion, extracellular matrix deposition, and cell expansion.

### Surface functionalization: liquid treatment

**FIG. 3.**
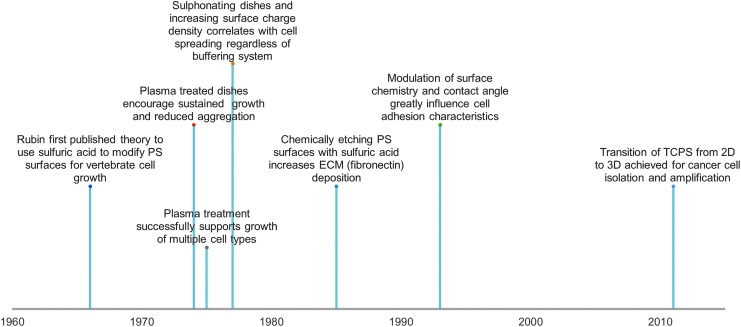
A brief history of the progression of TCPS. Initial articles described significant advances in PS as a culture material, with the basis for culture techniques largely occurring in the 1960s–1970s. 2D, two-dimensional; 3D, three-dimensional; ECM, extracellular matrix; TCPS, tissue culture polystyrene.

The first proposed mechanism for modifying the surface of PS to facilitate cell adhesion was introduced in 1966 by sulfonating the surface, with subsequent neutralization with sodium carbonate and water (Fig. 3).^1^ By the mid-1970s, the mass-produced TCPS dishes accepted today were becoming abundant in research with much interest in optimizing and upgrading the surfaces toward a general cell culture surface.^30–32^ Surface oxidation with strong acids began and continued to be used. Sulfuric acid treatment (20% v/v) not only aids in cell binding, but can potentially facilitate adhesion of proteins, such as fibronectin and vitronectin.^33^ The deposition of these proteins can mediate cell adhesion, spreading, and growth, primarily due to the surface receptors on cells present to bind certain proteins and act as adhesive agents.^27,34^ Other acids (e.g., nitric and hydrochloric) may show these benefits as well, but may tend to degrade surfaces, reducing the optical clarity.^33^

Although working with concentrated acids may not be desirable in a laboratory setting due to inherent safety concerns, safer liquid coatings are not preferable for creating uniform, stable, and permanent functional changes to the growth surfaces. For example, simulated body fluids can be used to facilitate growth of hydroxyapatite crystals on PS surfaces, with the goal of inducing osteogenic differentiation of mesenchymal stem cells.^35^ However, the surface density can be difficult to control, and they can be easily removed from the surface with applied force. As well, a protein coating or plasma-activated PS surface is required to deposit acidic residues and coordinate nucleation of hydroxyapatite crystals.^36^ Stable functional changes to the surface thus requires modification of surface chemistry using treatments that can enable oxidation or form stable covalent bonds. Full liquid immersion provides a technically direct method to introduce a number of surface chemistries (e.g., –CH_3_, –NH_2_, –SH, –OH, and –COOH) by selecting modifying liquids.^37^ Although this method may erode complex surfaces and printed geometry during modification, complete internal surface coverage is ensured. However, the use of plasma to enhance cell adhesion surface chemistry has become the convention for mass 2D TCPS production and remains popular in academic research. Liquid functionalization methods may reduce the optical clarity of surfaces, where plasma-based methods maintain translucency and provides chemical functionalization flexibility relatively easily.

### Surface functionalization: plasma treatment

**FIG. 4.**
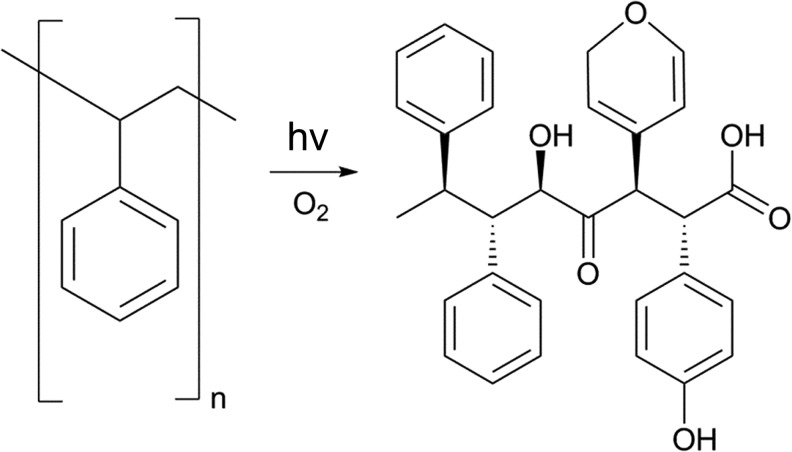
An example reaction demonstrating how oxygen can incorporate into polystyrene following a plasma treatment. The high potential of the reactive oxygen ions may break phenyl rings, or replace functional groups, changing the surface properties.

Plasma surface treatment remains the most prolific mechanism to modify PS over the past half century. Briefly, plasma surface modification occurs as current is passed across a gas, creating ionized species. Energetic ions may interact with the presented surfaces and incorporate or provide further functionality. Modifying the source gas effectively modifies the plasma composition and surface chemistry (Fig. 4).^38^ Plasma treatment, along with reactive ions, can produce electrons, free radicals, metastable species, ultraviolet (UV) light, and heat, all of which can work to deposit, etch, or chemically modify the surface of interest.^39^ The Falcon Plastics Company accidentally discovered the benefit of plasma treating PS for cell culture while attempting to prepare the surface for glass coating, however; it was found that the oxygen-containing plasma efficiently oxidized the surface and facilitated cell adhesion, ultimately leading to the preparation method for TCPS still used to this day.^3^ Early development of this approach looked at using glow discharge in vacuum and evaluating its effect on a number of cell types, where it was found that the surface treatment increased cell spreading and growth rates compared with untreated surfaces.^31^ Native PS surfaces are associated with early growth rate plateauing, likely due to reduced metabolic activity for self-adhered populations.^30^ Additionally, the negative surface charge encourages nonspecific surface absorption of serum proteins contained within the media, potentially mediating cell adhesion.^30^ There remains a number of confounding factors which make direct correlation of surface chemistry and cell interaction difficult, as surface charge, surface strain, media formulation, and cell type can all influence cell adhesion to surfaces. Even with these limitations, however, researchers have evaluated many excitation and functionalization gas combinations that have led to successful modification of PS surfaces.

The composition of the process gas and the plasma source configuration used to carry out plasma modification dictate the ultimate surface chemistry (Table 1), with gas combinations usually determined by the vacuum system chosen, power supply, level of purity required, and gases obtainable. The majority of research has involved modifying surfaces to incorporate oxygen- and nitrogen-containing species, with the objective of creating surface chemistry that encourages cell adhesion, proliferation, and functionality.^40,41^ Several studies have shown that increased plasma treatment time led to a higher oxygen concentration on the surface (i.e., increased wettability),^46,47^ but this alone was not a very strong determinant for cell adhesion and spreading.^48^ Interestingly, hydrophilic surfaces that displayed water contact angles between 40° and 60° appear to facilitate *in vitro* cell adhesion for human umbilical cord vein endothelial cells and HeLa cells,^49^ with the incorporation of carbonyl groups demonstrating the best adhesion characteristics.^28^ The breadth of process gases investigated, including a selection of organic compounds (e.g., acetone, methane, methanol, and formic acid) incorporated into an oxygen plasma have revealed some correlation between surface chemistry and cell adhesion rates.^42^ However, the mechanism of cell adhesion is a complex process, and variables are difficult to isolate. It is believed that the contributions other than surface chemistry are playing important roles in *in vitro* cell adhesion, for example the bulk polymer chemistry and presence of organic molecules,^42^ such as extracellular matrix proteins and serum components that may be inherently present in cell cultures.^43^

**Table 1.** Process Gases Used, Resulting Chemistries Found, and Major Study Impacts on Cells

*Gas combination*	*Surface chemistry (XPS determined)*	*Cellular impact*	*Reference*
Air	Carbon: 86.2%	Facilitated attachment under rotary conditions of L929 mouse fibroblasts	40
	Oxygen: 12.0%		
	Nitrogen 1.8%		
Ammonia (low pressure)	Carbon: 65%	Increased viability of human mesenchymal stem cells (122.7% increase in metabolic activity), human dermal microvascular endothelial cells (150.4% increase in metabolic activity) as compared with TCPS	41
	Oxygen: 5%		
	Nitrogen: 9.4%		
Acrylic acid (low pressure)	Carbon: 39.6%	Similar metabolic activity compared with TCPS	41
	Oxygen: 31.8%		
	Carboxyl: 17.0%		
Carbon dioxide (low pressure)	Carbon: 70%	Reduced enzymatic activity vs. TCPS (86.9%)	41
	Oxygen: 12.3%		
Argon (low pressure)	Not specified	Mouse fibroblasts found to have peak attachment density with short (<10 s) treatment times, and no difference between 10 and 30 s	28
Acetone, methane, methanol, formic acid, and oxygen	Varied with formulation	Hydroxyl groups do not correlate with cell growth of bovine aortic endothelial cells (*R*^2^ = 7.6%), carbonyl groups correlate better (*R*^2^ = 57%)	42
		Human umbilical vein endothelial cells found to adhere and grow on PS only with >17.7% oxygen content, matching TCPS	
Nitrogen or ammonia (10%), argon or helium (balance)	Carbon: 91% Oxygen or Nitrogen: 9% Varied with formulation	Find greatest cell attachment efficiency BCP-K1 cells with both ammonia and nitrogen dopant gases using helium plasmas. Greatest proliferation found for nitrogen/helium and ammonia/argon plasma-treated surfaces	43
Ultraviolet ozone	Oxygen: 36%	Chinese hamster ovary cells. See >80% of seeded cells attach under 3 hr incubation under 3 min of surface treatment, better than TCPS	44
	Find washing with water reduced the surface oxygen content.		
Ammonia plasma	Varied with sample	High cell affinity of human fibroblasts onto PS surfaces. Good amination of the surfaces with 15–20% of the total nitrogen content detected on the surfaces, with total amines presented increasing with increasing plasma intensity.	45

Significant work has been performed to understand the link between surface chemistry and cellular response. To date, it is difficult to find a unifying theory for all cell types, however, providing surface chemistry with a high degree of biomimicry (i.e., surface oxygen and nitrogen incorporated as carbonyl, carboxyl, amine, etc.) appear to improve cellular response during *in vitro* culture.

PS, polystyrene; TCPS, tissue culture polystyrene; XPS, x-ray photoelectron spectroscopy.

The absence of clear trends indicates numerous pathways for cell adhesion and spreading, and the selection of chemistries incorporated on the surface (e.g., carbonyl, hydroxyl, or carboxyl) may be necessary but insufficient for *in vitro* cell growth. For instance, one mechanism may require matrix proteins to absorb first through the interaction with the surface, which in turn allows cells to anchor down.^42,50^ One example of this phenomenon is thought to be initiated through the binding of extracellular matrix to a plasma-deposited amine surface, which then regulated the interaction and subsequent attachment of human mesenchymal stem cells.^43^ Although the mechanism remains unclear, cells may better recognize randomly adsorbed and often denatured proteins on plastic surfaces than the surface chemistry provided by plasma treatment, allowing cells to modify the surfaces and deposit their own extracellular matrix.^51^

An interesting effect of plasma treatment is the interaction of gas-phase ions with the PS surface, which has been reported to influence more than just the final surface chemistry. Plasma ion implantation and incorporation of free electrons can induce unnecessary charging on the surface,^52,53^ which may influence efficiency and extent of cell adhesion and spreading. This may also influence the type of serum proteins that absorb to the surface, which may subsequently regulate the type of cells that attach.^29,32^ In addition to the ion interaction with the surface, it has been demonstrated that the length of time the surface is exposed to the plasma, as well as the power applied, can have a significant effect on the surface chemistry and topography. For example, longer treatment times are associated with lower contact angles and higher surface free energy due to the attachment of oxygenated functional groups and breakdown of phenyl groups,^48^ which may be facilitating cell adhesion through enhanced oxygen or electron incorporation on the surface.^54,55^ As well, increasing the plasma source power tends to create more complex (oxide, nitride, hydroxyl) surface chemistry owing to the kinetic energy available for increased bond breaking and formation.^55^ The surface chemical surface facilitated with increased voltage can be accompanied by roughened surfaces, which have been shown to increase cell attachment, growth, and viability.^56–58^

Native PS surfaces are considered smooth (root-mean-square roughness of ∼1.7 nm).^44^ Plasma treatment tends to break substrate surface bonds and induce surface roughening.^59^ UV ozone exposure has been shown to leave surface pillars between (20 and 400) nm tall,^44^ which can possibly influence focal adhesion location and spreading.^60^ Surface wrinkling has also been observed with scanning electron microscopy and atomic force microscopy, with surface roughness generally increasing with increased energetic source exposure time.^59^ Aside from disrupting the otherwise pristine surfaces, the potential advantages of plasma treatment may be tempered by the longevity of treatments. Atmosphere or water exposure has caused some prepared samples to deteriorate over time (potentially removing up to half of the bioactive residues), necessitating sample storage under nonreactive gasses (e.g., Argon) before use.^61^ Although the functionality on the surface is preserved, shelf stability is necessary to appeal to a mass production environment. Oxygenated surfaces appear to be the exception, as samples have been shown to maintain surface chemistry for >1 year.^42^

In cases where site-specific surface modification is required, plasma jets can be directed through a shadow mask^62,63^ or surfaces can be partially covered with photoresist resin^64^ to provide spatially distinct regions for surface modification. Another approach is to use focused plasma treatment from a dielectric barrier discharge jet, by confining the jet with glass capillaries as small as 100 μm in diameter, modifying regions up to 1.5 mm in diameter, even when using atmospheric low-flow-rate plasma.^65^ This approach effectively localizes cell growth, which is beneficial for isolating and patterning cell populations.^66^ The area modified by the plasma beam is not limited to the size of the striking plasma jet, as reactive species follow the gas flow profile away from the impingement point and modify the PS surface for some distance. Given these limitations, oxygen plasma has been used to route and pattern cell constructs, localizing cell adherence.^67,68^ Further effective means of localizing plasma treatment for cell culture requires modifying the characteristics or geometry of the substrate. For example, treating 3D objects in a layer-by-layer fashion or treating fully fabricated objects at the completion of fabrication could both effectively modify complex objects and deposit custom surface chemistry.

**FIG. 5.**
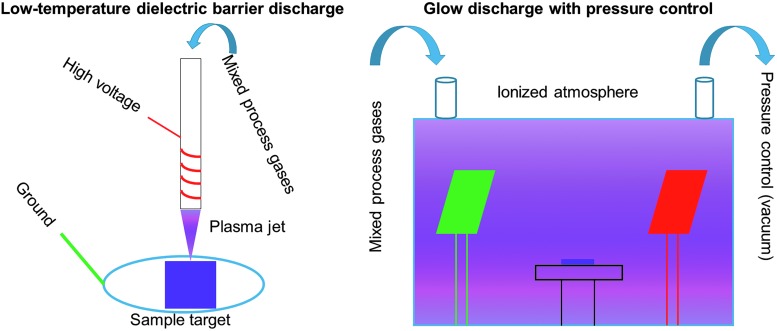
Major division of plasma treatment apparatuses. *Left*. A dielectric barrier discharge system is described, where gas is ionized and directed toward a target substrate. Incorporation of this approach with a 3D printing or electrospinning technique could see directed functionalization on individual fibers within a larger construct. *Right*. The use of a chamber in a glow-discharge system allows for greater pressure and gas composition control, as the reactive species are fully contained. Ionizing the entire atmosphere may better treat the target as well.

Treating 3D objects with plasma remains a challenge. To confine the treatment, immersion ionization or a confinement chamber at low (<100 Pa),^64,69^ medium (∼1 kPa),^70^ and atmospheric (∼100 kPa) pressures^55,65,71^ have been used (Fig. 5), reducing safety concerns brought with corrosive components. By controlling the atmospheric pressure, one can provide better control of the treatment environment and ionize the entire volume, but this methodology requires additional equipment. This can be overcome by custom-made low-temperature dielectric discharge setups; however, it requires the ability to direct the jet through the bulk of the object. Combining these treatment systems may be the best course yet to fully modify an interconnected surface, and best balance the safety, cost, and research goals.

### Surface functionalization: future methods

Passive means to facilitate cell adhesion have laid the foundation for cell culture. The means to provide specific chemical functionality to the PS surfaces is well investigated, and the variety of applications continues to be explored. With the range of functional moieties possible, the next steps should look at mechanisms to target specific cell types and means to sequester and expand these cells. The same mechanisms which functionalize PS surfaces can be used as a base for advanced grafting techniques (such as self-assembled monolayers^72^ or polymer brushes^73^). Grafting of poly(N-isopropyl acrylamide) to TCPS surfaces has successfully released adherent culture cells, without introducing additional enzymes, by inducing a conformational change in the polymer brushes as the culture temperature passes below the lower critical solution temperature.^74^ Multilayered rabbit epithelial corneal^75^ and neonatal rat cardimyocyte^76^ tissues have been grown *in vitro* and transplanted back into their host species, maintaining cell–cell junctions, deposited extracellular matrix, and functionality of the tissue. Plasmas containing argon and/or oxygen have been used to aid in the grafting of additional chemical species, such as *N*-vinyl-2-pyrrolidone, to improve biocompatibility, adhesion, and proliferation of L929 cells.^77^ By selecting modifications which target or bind specific cells, successful coculture or filtration could be possible. DNA has been grafted to PS surfaces using secondary amines,^78^ a technique that could be translated to antibodies as well.^79–81^ Additionally, glucose has been sequestered to PS surfaces using thiol-ene “click” chemistry,^82^ a mechanism which could be further investigated for advanced surface functionalization. These approaches open the possibility for selective growth surfaces and localized coculture on single dishes, but the stability of such surfaces must be investigated to enable large-scale acceptance and adoption. Plasma preparation of surfaces can more efficiently prepare surfaces to accept patterning, without the need of stamps,^83^ extensive photolithography preparation,^84^ and the ability to treat large surfaces and 3D objects, something difficult to achieve with microcontact printing.^85^ Selecting copolymers can also aide in effective cell and protein adhesion regulation. Further exploration of “smart” surfaces is warranted to enhance tunable and selective culture techniques and to develop niches for specific cell types and interactions. Inkjet printing has been used to sequester cells to specific locations on PS surfaces, a step toward direct spatial patterning on proven *in vitro* growth surfaces.^16^ Combinatorial screening of bioactive molecules printed or conjugated to surfaces could be used to investigate complex cellular pathways by decoupling and determining how multiple proteins impact cellular processes.^86,87^ All the chemical changes possible necessitate taking advantage of the numerous means to create complex growth platforms, looking at methods to mimic the body. While chemical cues are often necessary to elicit a functional response in a tissue or target cell population, the geometry presented to the cells are often just as important and warrant discussion as well.

## Fabrication Methods of 3D PS Growth Platforms

### Motivation for 3D platforms

**FIG. 6.**
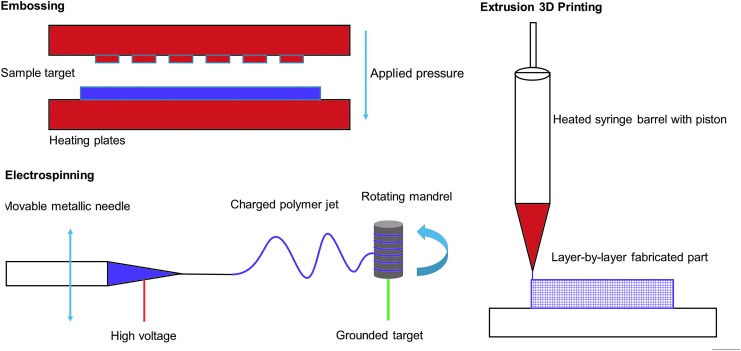
Fabrication approaches. *Top Left*. High heat and pressure can be used to mold the material into highly structured shapes with hot embossing/injection molding approaches. *Bottom Left*. Applying a high voltage potential between the mandrel and polymer-containing syringe, electrospinning creates fine structures and repeated rotations can build dense, sizable meshes. *Right*. 3D printing offers an excellent balance between fabrication control and achievable detail, but little work has pursued PS as a 3D printing base for cell-based work.

The widespread adoption of standard flat cell culture dishes over the last half century has driven *in vitro* cell culture and research. TCPS dishes certainly serve their purpose, and biologically based research would not be developing cutting-edge technologies without them. However, transitioning culture from a 2D to 3D substrates could improve the biomimicry, thus improving cell–cell interactions and increasing the efficiency of *in vitro* cell culture. Fabrication techniques, specifically casting, electrospinning, and 3D printing, seek to solidify this transition (Fig. 6). As discussed earlier, casting is often the easiest method to produce cell scaffolding, however, the production of complex 3D microstructures is limited.^16^ Electrospinning can create highly porous, interconnected objects, but they are difficult to control, produce, and manufacture reproducibly.^88^ Three-dimensional printing balances casting and electrospinning approaches, however, often requires expensive or custom fabrication equipment to achieve research goals.^89,90^ Recently, 3D printing technology maturation continues to decrease equipment costs and increase flexibility of material choice.^91,92^ Fabricating and functionalizing microspheres provide a unique path for high-density adherent cell culture in suspension, but the applications may be limited to cell types that are able to withstand the mechanical forces in the dynamic culture system.^93^ An ideal scaffold fabrication method would balance the resolution, speed, accuracy, and cost of these approaches.

Transitioning TCPS from a 2D substrate to 3D, regardless of the selected fabrication strategy, offers significant benefits. The potential exists to revolutionize cell culture: 3D models have been shown to improve disease and pharmaceutical modeling^94–97^ and capitalize on dynamic culture methods, generating clinically relevant geometries and numbers of cells.^98–100^ Transitioning culture from a 2D to a predictable 3D model standard would drastically increase the biomimicry of *in vitro* culture methods. These do come with additional challenges: ensuring sufficient nutrient exchange through the bulk of the object (overcome with bioreactor expansion methods^101^), visualizing growing cells (overcome with utilizing a clear material, such as PS, or with a microfluidic approach^16,102,103^), and efficient capture of the cells after culture and expansion (overcome with highly permeable, porous, and interconnected scaffolds^104,105^). Transformation of PS to a 3D culture substrate would allow continued investigation into the influences of geometry^106^ and porosity^107^ on *in vitro* cell growth, while utilizing a proven and versatile growth substrate. In the following section, we survey these fabrication methods and provide some perspective on continuing to advance PS as a universally accepted culture surface.

### Fabrication: electrospinning

Electrospinning remains a lucrative fabrication method to produce finely structured cell culture substrates. By manipulating the interactions between solvent, polymer, and current, electrospinning can form fine polymer strands to fabricate mesh structures. Where applied voltage and PS solution content have obvious influences, solvent choice impacts many solution parameters dictating PS electrospinning success (i.e., dipole moment, conductivity, boiling point, viscoelasticity, viscosity, surface tension, and density).^108^ Solvents with high dipole moment [(5.3–12.7) × 10^−30^ C*m] and moderate conductivity [(0–3.7) × 10^−4^ S/m], such as 1,2-dichloroethane, dimethylformamide, ethyl acetate, methyl ethyl ketone, and tetrahydrofuran, appear to reduce the “bead-on-a-string” morphology, producing uniform fibers, therefore, contributing to electrospinning sucess.^108^ Additionally, reducing polymer content in PS solutions generates meshes without surface defects or beaded fibers, and increasing the conductivity of these low-concentration solution tends to stretch the polymer jet better, reducing fiber diameter by an order of magnitude (down to several hundred nm).^88^ To ultimately grow cells in high density in these platforms, studies investigating the effect of fiber alignment on cell attachment and morphology have been conducted on these highly packed, fine fiber as an amenable cell culture platform. (A more complete review of electrospinning to create cell scaffolding has been provided by Boudriot *et al.*^109^).

Cells are known to align along individual fibers, potentially due to the alignment of polymer chains within the larger microfibers.^110^ PS microfiber meshes have been used to align and grow MC3T3 cells along the fibers, where osteoconduction was observed, therefore potentially utilizing PS scaffolds as the base for a bone scaffolding substitute. Keratinocytes and endothelial cells appear to organize themselves 3D, layering in native epidermal–dermal structures along air–liquid interfaces, demonstrating intercellular signaling within the electrospun scaffolds.^111^ In addition to polymer chain alignment, the porosity of the mesh architecture also influences the morphology of the adherent cells contained within the construct. Highly porous electrospun PS facilitates human induced pluripotent stem cells to develop 3D aggregates, allowing for cell migration and signaling within a contained 3D object, and accelerating the shift to a bioreactor model with minimal external manipulation for culture, reducing or eliminating outside manipulation while maintaining pluripotency.^112^ The highly porous and interconnected nature of electrospun fibers are an appealing substrate for surface functionalization, as they allow for relatively large (>1 cm) scaffolds with micron and nanoscale inner structures to be fabricated within hours and custom functionalization to suit specific research needs.^113^ The ability to physically absorb bioactive molecules, therapeutic agents, and modulate the surface chemistry and piezoelectric properties of these interconnected, high surface area objects make highly porous electrospun meshes intriguing for tissue engineering, drug delivery, and other biomedical applications (a more complete review of functionalization approaches of electrospun objects has been written by Yoo *et al.*^114^).

The development of electrospinning technologies for PS has revealed several drawbacks, most notably the difficulty in defining and controlling fiber placement. The randomness associated with electrospun objects and random instability in the electrospray process reduces repeatability of experiments. Ambient humidity and PS molecular weight have been found to influence the development of surface pores, further reducing the uniformity of the strands and the structural integrity of the final polymer network.^115^ Ambient humidity can interfere further by inducing the formation of surface winkles in the final PS mesh, which, along with voids in the bulk of strands, can be removed through annealing, adding additional processing steps, which could harm the delicate structures formed.^116^ From our ongoing work with 3D-printed PS, environmental influences, such as ambient humidity, appear to have little influence on fiber morphology, so long as the base material is stored properly. The substantial influence of environmental conditions in electrospinning necessitates highly controlled environments for the work, or significant time investment to achieve morphologically expected scaffolds. The above reasons make more directly controllable methods, such as 3D printing, appealing, often allowing for greater environmental flexibility and control over the fabricated object.

### Fabrication: 3D printing

A significant need exists to fabricate customized structures with biological relevance, and 3D printing is emerging as a leading technology to achieve these goals. Over the last several years, various 3D printing technologies have moved within reach of even the most casual researcher. However, PS has generated little research interest to date as a 3D printed, cell-contacting growth material. This likely stems from the difficulty in liquefying PS without thermally degrading PS, as the polymer structure will break down before transitioning fully to a true liquid from a solid.^117^ Overcoming this obstacle requires specialized equipment to extrude the semisolid. In addition, fine fibers of PS are mechanically weak. These limitations often limit PS's application as a primary structural element in 3D printed constructs, relegating PS to serve as a coating. For instance, optically clear support materials, such as Vero Clear, can be printed to form channels with an oxygen plasma-activated PS coating.^118^ This allows for both specific surface functionalization and direct scaffold control. These methods facilitate interactions between printable materials and cells by utilizing chemical interactions known to be amenable to cells. However, emerging evidence demonstrates the ability to directly 3D print PS,^119^ making these grafting approaches obsolete.

Commercially available products now exist using 3D printed, plasma-treated PS to grow and isolate cancer cell lines, with potential applications in general cell culture.^119^ Three-dimensional printing provides a method to design and test culture surface geometry tailored to specific cell environments. By utilizing computational modeling to quantify surface shear stresses, oxygen content, and mechanical stresses,^120–124^ there remain many possibilities for *in vitro* cell culture with targeted biological applications (e.g., the bone marrow niche). By coupling relevant biological regulators with large and internally complex surfaces, one would be able to grow large number of cells on biomimetic objects.

The evidence is present to establish the feasibility of complex 3D printed PS parts to become scaffolding for cells and harnessing the same surface functionalization methods employed in 2D. Three-dimensionally printed PS should become a mainstay for cell culture, allowing for concentrated culture volumes, dynamic culture environments, and complex surface chemistry to dictate cellular interactions for *in vitro* study. Unfortunately, the financial barrier to 3D print scaffolds leads researchers to seek other methods to capitalize on dynamic culture approaches. A highly sought approach incorporates individual beads to encapsulate cells, either fused together generating a single larger object,^125^ or circulating within the culture media. Microsphere carriers provide an alternative to structured scaffolds, allowing direct surface customization and dense cell culturing.

### Fabrication: microspheres

Microcarrier culture aims to suspend beads on the order of (0.01–1) mm in media and maintain the culture suspension with a bioreactor.^126^ Cells can adhere to the bead surfaces and remain suspended to increase culture density and utilize similar advantages of other bioreactor cultures (e.g., shear stress, oxygen content, and geometry effects). These microspheres can be fabricated directly with a microhead^127^ or in solution through dispersion polymerization, with the ability to control the particle size and molecular weight.^128^ Microspheres remain an appealing option for cell culture: the high density of suspended carriers and scalable volumes effectively increases culture area to yield large numbers of phenotypically expected adherent cells.^129^ Particularly, PS microspheres have been successfully fabricated through the miniemulsion process, yielding PS carriers as small as 115 ± 9 nm with both carboxylic and amine tags, to activate proinflammatory responses in human macrophages.^130^ The versatility of fabricated and commercially available beads remains unparalleled. As a base growth platform, PS microspheres can be functionalized using corona discharge or induced to carry a positive surface charge.^93^ As a core carrier, PS microbeads can be coated to carry proteins,^131^ glass,^132^ or peptides.^129^ PS microbeads functionalized with quantum dots and magnetic nanoparticles efficiently facilitate capture and specific population enrichment.^133^ Targeted functionalization of PS microspheres could be used to guide cell differentiation in large-scale bioreactors without modifying the media composition, taking advantage of known chemoresponsive cell types, as previously performed with other base materials.^134^ Carboxylated PS has been shown to create a surface with high epithelial cell attachment efficiency on static microbeads.^135^ In addition, PS microbeads make excellent carriers to facilitate cellular uptake to deliver exogenous cargo,^136,137^ DNA vectors,^138^ monoclonal antibodies,^139^ or for localizing within tumor spheroids.^140^ However, a major drawback to microcarrier culture, can be the clustering of the cell carriers, resulting in aggregated spheroids rather than distinct circulating populations, poor attachment efficiency, and the difficulty in retrieving grown cells.^141,142^ To diminish these drawbacks, additional culture steps are necessary, such as cell suspension filtration to separate carriers from cell populations during harvest.^143^ While the flexibility of microbead culture, from modulating surface charge to conjugating proteins through (1-ethyl-3-[3-dimethylaminopropyl)carbodiimide hydrochloride]) chemical linking has been found to help facilitate binding and attachment,^144^ the presented limitations reduce the clinical applicability of microbead systems.

## Conclusion

PS has been thoroughly explored as a useful cell cultivation tool for decades, but the applications of the polymer may be just starting to be harnessed. The proven cell culture substrate shows promise for future and continued use, but needs to be upgraded for the current challenges in the 21st century. For too long, researchers have relied on basic oxidized surfaces, where the possibility has been demonstrated to create specialized surfaces to select distinct cell populations or phenotypes. As the understanding of cell culture improves, so should the substrates used to grow the cells, becoming more specific and targeting individual populations.

As cell culture transitions from 2D to 3D substrates, so too should the most basic designs of cell culture platforms develop. A wealth of knowledge is out there, waiting to be tapped into including basic scientific understanding of how cells attach and regulate growth *in vitro* and why certain residues are more appealing to cell culture. Translating these to applied research could look at functionalizing materials to yield specific responses (e.g., directing differentiation of stem cells). There exists a wide range of possible research questions, tackling this most basic of culture surfaces. The only question left is: where is PS going as a culture surface?

As tissue culture continues to evolve, so should our surfaces. Whether this is specific chemical or recognition moieties, or 3D environments to best harness the power of *in vitro* culture to mimic a cell microenvironment, treating culture surfaces to specifically act for a given cell type provides a course for personalized medicine and directed cell growth. TCPS provides a cost-effective means to grow a variety of cells, but better, equally stable, materials continue to be investigated to shrug the ‘one-size-fits-all’ approach to *in vitro* cell culture. The optical clarity and relative cost make PS a unique material to spearhead these efforts to customize surfaces. The presentation of phenyl rings provides many locations to facilitate cell-focused functionalization, and the low crystallinity of the material allows for relatively low processing temperatures. This combination of material functionality and formability is unique for a culture substrate, and likely holds many opportunities for advancing cell culture beyond the 50-year-old flat, traditional culture dish.
